# Comparison of Two Evacuation Shelter Operating Policies and the Role of Public Health Nurses after the Great East Japan Earthquake: A Qualitative Study

**DOI:** 10.3390/ijerph17228310

**Published:** 2020-11-10

**Authors:** Hiroko Mori, Shuichi P. Obuchi, Yasuhiro Sugawara, Takeo Nakayama, Ryutaro Takahashi

**Affiliations:** 1 Human Care Research Team, Tokyo Metropolitan Institute of Gerontology, Tokyo 173-0015, Japan; obuchipc@tmig.or.jp (S.P.O.); arawagus@outlook.jp (Y.S.); hibridge2002@ybb.ne.jp (R.T.); 2 Department of Health Informatics, Kyoto University School of Public Health, Kyoto 606-7501, Japan; nakayama.takeo.4a@kyoto-u.ac.jp; 3 Tamadaira-no-Mori Hospital Tokyo, Hino 191-0062, Japan

**Keywords:** operating shelter, disaster response, the Great East Japan Earthquake, public health nurses, qualitative research, operational period, human resource allocation

## Abstract

This study describes shelter operations by public health nurses (PHNs) in Kesennuma City, located near the epicenter of the Great East Japan Earthquake, which occurred on March 11, 2011. The data were semi-structured interviews with 10 PHNs, 2 nutritionists, and 2 general administrators conducted from July 2013 to January 2014. All transcripts were analyzed using the constructivist grounded theory approach. We identified two operating methods for shelters: shelters stationed by PHNs in the Old City, and shelters patrolled by PHNs in the merged district. These methods were compared using four themes. In emergency situations, “operational periods,” a predetermined short term for a leader to perform his/her duties responsibly, could be adopted for relatively small organizations on the frontline. PHNs must not only attempt to operate shelters on their own but also encourage residents to manage the shelters as well. Moreover, human resource allocation should be managed independently of personal factors, as strong relationships between shelter residents would sometimes disturb the flexibility of the response. Even when a situation requires PHNs to stay in shelters, frequent collecting of information and updating the plan according to response progress will help to maintain effective shelter operations.

## 1. Introduction

The 9.0-magnitude Great East Japan Earthquake occurred on March 11, 2011 and is the fourth-largest earthquake to occur in world history since the end of the nineteenth century [1]. This earthquake caused massive tsunamis, over 30 m high, leaving more than 18,000 people dead or missing [2]. Recently, enormous natural disasters have been regarded as public health emergencies alongside acts of terrorism, epidemics, diseases, etc. [3,4,5]. Many countries maintain permanent organizations that manage national disaster management system for disaster risk reduction (DRR), such as the Federal Emergency Management Agency (FEMA) in United States [6], the Civil Contingencies Secretariat in the United Kingdom [7], and the Civil Contingencies Agency in Sweden [8]. These government agencies are responsible for initiatives and measures and for cooperating with other ministries, local governments, and regional communities. The European Union developed the Civil Protection Mechanism rescEU in 2019 to address emergencies that overwhelm an individual country’s ability to respond on its own [9]. On the other hand, in Italy [10], Germany [11], and Japan [12], the countermeasures initiative bodies are on the municipal, prefecture/state, or national level, depending on the scale of the disaster. While Italy and Germany each have a permanent ministry dedicated to disaster relief, Japan has only an ad hoc Disaster Management Headquarters in its cabinet for when a catastrophic disaster occurs. The minister of this ad hoc department is selected from among other, existing ministers and holds a double appointment. To enhance community resilience to disasters, citizens and multidisciplinary professions are expected to contribute to reducing disasters’ adverse influence. FEMA developed a nationwide approach to volunteer training [13]. In Italy, more than one million disaster relief workers such as cooks and truck drivers provide services for a fee in evacuation shelters, utilizing their professional skills [14]. As an international agenda on specified natural disasters, the Sendai Framework for Disaster Risk Reduction 2015–2030 [15] proposes that each country take primary responsibility for DRR and establishes the following four priority policies: understanding the disaster risk, strengthening efforts, investing in DRR to ensure resilience, and disaster preparedness for effective response and recovery. Using these priorities as common international criteria, several researchers have evaluated DRR projects that have been developed independently in different countries [16,17,18]. This framework also provides a useful paradigm within which to shape the research field’s strategic development. The WHO’s Thematic Platform for Health-EDRM [19] was established, and five primary research areas on DRR were identified by an international research network [20].

When a disaster occurs, the Incident Command System (ICS) is typically used for disaster risk governance. The ICS, a standardized management system for disaster and incident sites, was developed in the United States in the 1970s. The system standardizes how ICS organizations are formed, organization names, Incident Action Plan formats, and the methods of communicating appropriately and efficiently in crisis situations. The target incidents can range from daily incidents to incidents requiring crisis management, such as terrorist incidents, regardless of the type of disaster [21]. An Incident Action Plan also consists of repeated operational periods during which one commander will carry out his/her duties responsibly. For evacuation decision-making plans adopted in Australia [22] and the United Kingdom [23], operational manuals have been prepared and the importance of the role of public health nurses (PHNs) in evacuation shelters is recognized. In Poland, when meteorological phenomena carry the risk of immediate threat, the Government Centre for Security Alert sends personnel to potential dangerous area [24,25]. On the community level, the Los Angeles County Community Disaster Resilience project provides a citizen education program for DRR through which awareness of community disaster resilience is improved by “transforming disaster planning and response from just ‘me‘ to include ‘we’“ [13]. PHNs are expected to play a crucial role in citizen education through this project as well.

In Kesennuma City located near the epicenter of the Great East Japan Earthquake, the municipal government immediately established a Disaster Response Headquarters, and PHNs began to provide healthcare services in evacuation shelters. In the acute phases of natural disasters, evacuation shelters serve several important functions, including securing residents’ safety, providing a hub for the transmission of official information, and acting as a location where health and social services can be coordinated [26]. The Sphere Project proposed guidelines for improving the quality of shelters under critical conditions [27]. However, the performance of PHNs under disaster conditions was not mentioned in detail. Although some studies reported on the challenges PHNs face in evacuation shelters with regard to health problems and behavior towards evacuees [28,29,30], to our knowledge, no study has examined the performance of PHNs in evacuation shelters and shelter operational policies.

The present study aimed to describe the different roles of PHNs in evacuation shelters over time after a disaster and to grasp the implications of their activities on their own. Before the Great East Japan Earthquake, national and local government regulations did not outline how PHNs should work in shelters in Japan. Therefore, this study describes how the PHNs behaved in the “extremely special situation” that manifested after one of the world’s largest earthquakes and accompanying devastating tsunami. In Kesennuma, reflecting the influence of recent mergers (2006 and 2009), the administrative PHNs’ approach to operating the shelters may be different in the acute phase of disasters. It is extremely meaningful to record this valuable human experience as common intellectual property.

## 2. Materials and Methods

### 2.1. Data Collection and Analysis

Interviews using an interview guide (Appendix A Table A1) were conducted in a longitudinal project as the first phase. The subjects were professionals in the medical and welfare fields and administrative officials. The second phase involved selecting nine survey patricians from the first phase. The survey’s topic in the second phase focused on issues facing Kesennuma in the five years since the earthquake. The purpose of the longitudinal project was to create an archive of data on how professionals in the medical and welfare fields and administrative officials struggled to carry out their work in the disaster response phase, despite being themselves victims of the earthquake. Immediately after the earthquake, various health surveys and studies were planned in the disaster area by people from other areas. Some of the studies did not heed ethical considerations and placed a heavy burden on the involved residents, and the Japanese Government as a result issued an unusual alert to researchers [31]. In that situation, the Tokyo Metropolitan Institute of Gerontology signed a contract with Kesennuma municipality in January 2012 and began this longitudinal project, named the “Study on the fulfillment of professional responsibilities in the medical and welfare fields in the aftermath of the Great East Japan Earthquake.” A researcher (YS), with connections to the local community performed theoretical sampling over the telephone and through in-person visits. The interviewers had no relationship with the interviewees prior to the study. Informed consent was obtained before the interviews. The interviews were recorded using a voice recorder, and the interviewers immediately wrote down the content of the interviews using codes as the initial analysis. Kesennuma municipality confirmed the study protocol before its implementation and cooperated with the recruitment of research subjects. The research plan was approved by the Tokyo Metropolitan Geriatric Hospital Ethics Committee (Project identification code: 25-513). The results of this project were published as a research report with narrative data and as a book for sale to the general public. 

Among the data obtained in the first phase, we focused on the interviewees who supported the evacuees in evacuation shelters for our research as the secondary analysis. Although we gained the outlines of different PHNs’ roles in the evacuation shelters, the details of their activities and the meaning-making their roles on their own were unknown. Anonymized text transcriptions were analyzed using the constructivist grounded theory approach [32]. The analytical process was audited by researchers who were not involved in the analysis. For the qualitative analysis, we used ATLAS.ti version 7.5.12 (ATLAS.ti Scientific Software Development, Berlin, Germany).

### 2.2. Damage to Kesennuma Following the Great East Japan Earthquake

**Figure 1:**
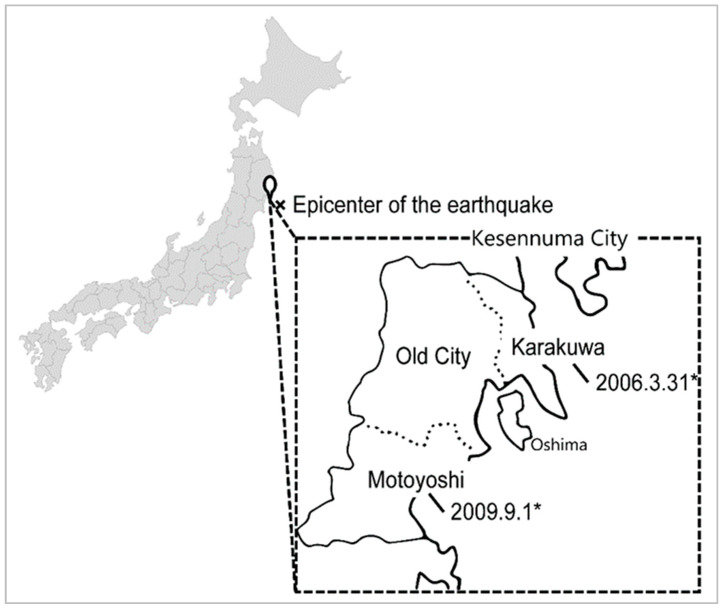
Locations of the three districts that merged to form Kesennuma City. * Date of mergers with the Old City.

In the initial phase of the disaster, tsunamis extending than 10 m in height struck four times during the 3 h following the earthquake. As a result, 20% of the planned area of the city was flooded, almost every building in the town collapsed, and a large amount of debris and mud accumulated [33]. Kesennuma City is formed from the recent mergers of three districts (in 2006 and 2009). At the time of the earthquake, the administrative culture from before the merging remained intact. The former urban area, which is known as the “Old City,” was merged with the “Karakuwa District”, and the “Motoyoshi District.” The names of both these districts are the names of the respective villages from prior to the merger. The current Kesennuma City is comprised of these three areas (Figure 1).

In the Old City, where the municipal office was damaged by the tsunami, 23 heavy oil tanks on the banks of Kesennuma Bay caught on fire. The fire then spread through the city and continued for 12 days. More than 100 evacuation shelters were opened, and the most crowded shelter housed more than 2000 evacuees. These numbers fluctuated over the 20 days following the disaster, gradually decreasing over the weeks leading up to April. The traffic infrastructure in the three districts was destroyed, so each district had to respond to the disaster independently during the acute phase of the earthquake. The demographic data and the extent of the damage in each district, excluding the local islands, are shown in Table 1 [33,34].

The proportion of people aged 65 and over in each district’s population was approximately 30%, which was higher than the 23.1% in Japan as a whole. The population density of a square kilometer ranged from 175 to 101 in the 3 districts, while the population density of the 11 square kilometers in the center of the Old City was 1781 [33]. This population density per square kilometer was similar to that of Houston, Texas in the United States [35].

## 3. Results

### 3.1. Participants and Data Collection

In the first phase of our longitudinal project, 79 interviews were conducted from July 2013 to January 2014. The second phase occurred from November to December in 2016. Each interview was performed by two researchers at each interviewee’s workplace. When the number of interviewees in the first phase exceeded 70, we checked the data collection status for each of the three districts and specialized fields for theoretical sampling [36]. Theoretical sampling is defined as a process of data collection and analysis where the next data should be collected after the previous data has been analyzed in the initial stage [37]. As a theoretical saturation, we confirmed that the collected data provided sufficient information and did not require new data [36]. For the purpose of this study, of the 79 interviewees, we selected individuals who worked evacuation shelters, leaving 15 research subjects for our secondary analysis. The selected interviewees included 11 administrative PHNs, 2 nutritionists working in the city government, and 2 administrators in charge of the community development and health and welfare departments. The average duration of the interviews was 1 h and 29 min (maximum: 2 h and 45 min; minimum: 1 h). 

### 3.2. Analyzed Districts and the Two Shelter Operation Policies 

The analyzed data were limited to those from Motoyoshi District and the Old City. Eight of the interviewees were from the Old City, two were from the Karakuwa District, and five were from the Motoyoshi District (data belonging to the district were from the onset of the disaster). However, one week after the disaster, a PHN from two interviews in Karakuwa District had moved to the Disaster Response Headquarters in the Old City. The data from Karakuwa District were thus insufficient for an adequate analysis. The narrative data of the PHN who moved from Karakuwa were analyzed as a part of the data from the Old City. In 2011, 21 PHNs were working full-time in Kesennuma City [38], representing 47% of our study subjects (10/21), and leaving us a sample size of 14. Using the constructivist grounded theory approach, two different operational policies and four themes regarding the allocation of PHNs in the evacuation shelters were identified: stationing in the Old City and patrolling in Motoyoshi District. These districts shared many factors (e.g., local ties, cultural factors, and climate); however, two divergent approaches to shelter operations were adopted due to disruptions in the communication and transportation links between them.

### 3.3. Description of the Operating Policies Using Four Themes 

The two operational policies were compared using four themes: (1) the lack of an operational preparedness policy for the shelters, (2) the provision of support to the shelter residents, (3) the autonomy of the shelter residents, and (4) the relationship with the external PHN support teams. The narrative data are shown in Table 2.

#### 3.3.1. Theme 1: The Lack of an Operational Preparedness Policy for the Shelters: The Onset of the Disaster

Before this earthquake, the two types of operational policy had not been finalized, and no decision had been made as to how PHNs should work in evacuation shelters. Therefore, when the disaster occurred, the PHNs had to respond promptly. The decision process regarding the allocation of the PHNs differed regarding whether their opinions were reflected in the policies.

##### Station Type

In the Old City, at the onset of the earthquake, everyone in the municipal offices evacuated to the parking area on the hill behind the office, and they witnessed the spectacle of the tsunami’s waves crashing into the municipal office building. Several hours after the waves had receded, the Disaster Response Headquarters of Kesennuma, based in the Fire Department Building, announced in the presence of all of the administrative officials that all PHNs and nurses should be stationed at 10 large-scale shelters. Detailed management plans for the evacuation shelters were not established before this earthquake, and the decision-making process did not seem to incorporate the PHNs’ views sufficiently.

- The operating method of each shelter was different. Id 02
- If an operating shelter system had been created, I think, the response to the disaster during March would have been a little better. Id 13
- We were waiting for commands from headquarters without knowing what to do. I don’t remember what time it was, but, at night, the headquarters’ command was for the public health nurses to support the shelters. Id 03

One participant felt that the policy had already been settled at the Disaster Response Headquarters and that there was no room for discussion. At this point, no one had information on the situation at the shelters or about the damage to the city. Therefore, the PHNs’ roles in the evacuation shelters were self-determined when they arrived at their requisite shelters.

- Everyone gathered in the city office’s large hall... we received a command from headquarters to move to an evacuation shelter. We were told to scatter to each of the evacuation shelters. Id 02
- We wondered, “When we do go out there, what should we do?” Id 01

Each PHN was assigned to an evacuation shelter based on territorial connections (e.g., the PHN’s place of residence). Support teams for the evacuation shelters were formed with one nurse and several administrative officials, who together assumed responsibility for the shelter. Although these teams immediately went to their assigned shelters, some of the PHNs were recalled to headquarters the following morning to be reassigned to shelters where a large number of elderly persons were gathering.

##### Patrol Type

Administrative officials at the Motoyoshi branch city office sought refuge on the roof, where they witnessed houses, cars, and other debris being washed away by the tsunami. Although the means of transportation to and communication with the Disaster Response Headquarters in the Old City were disrupted, the branch office building was not damaged, and administrative activities continued throughout the acute phase of the disaster. At the time, four PHNs and two nurses were working in the Motoyoshi branch. When a strategy was discussed for the PHNs’ performance in the evacuation shelters, the chief clerk of the PHNs recommended the PHN patrol policy to the community health policy leader in that district. The chief PHN thought that the resources of the available PHNs would be insufficient for the situation’s extensive damage. This clerk’s recommendation was immediately accepted and shared with all of the administrators, because all human resources in the branch office were on the same floor.

- No one can stay in the evacuation shelters. We will move around the shelters with two-person teams,” so I said and we decided. With that, a patrol-type evacuation shelter management style was established. Id 11
- Headquarters was in this large room. The self-defense force, firefighters, the fire brigade, and our desks were gathered on the same floor of this branch. All issues and decision-making procedures were shared with everyone on the floor, helping to understand the priorities in the need for assistance. Id 11

#### 3.3.2. Theme 2: The Provision of Support to the Shelter Residents: Approximately 72 Hours after the Disaster

During the survival phase of a critical disaster, administrative officials must manage evacuation shelters as the core center for life support, information, and logistics in a community. However, a wide variety of management tasks occur one after another, changing from moment to moment, and those involved can be overwhelmed by the situation. Under such conditions, one of the most important issues for PHNs might be to remain independent of these massive tasks and attend to health issues. The tsunami divided people into those facing life or death. Many of the people who had escaped from the risk of death were not injured. Most of the residents in the shelters complained about the lack of medication for chronic diseases as their main health problem.

##### Station Type

The PHNs from the severely damaged municipal office were not able to bring any medical equipment. Of course, they had no personal belongings such as a change of clothes.

- I couldn’t bring anything for healthcare equipment at all. I managed to contact my parents’ house and bring my parents’ blood pressure monitor. The only medical tool that can be used is the first aid kit provided in the public hall. Id 05

Moreover, the support team members had to explore the needs of the shelters for which they were responsible. Even in the Old City, the damage situation varied, and the problems facing each shelter were different. For example, some of the shelters reported deaths from hypothermia because of the flooding, while others struggled to accept hundreds of comparatively healthy residents rushing to find accommodation. In many of the shelters, the first responsibility of the PHNs was to create lists of the evacuees. However, many evacuees moved between shelters in search of their families over several days; therefore, creating the lists became unexpectedly laborious. Despite receiving a large amount of supplies from the Disaster Response Headquarters of Kesennuma, few instructions were given regarding the shelters’ operating policies or the damage throughout the city.

- [When I was reassigned to the shelter the next day] an officer at the headquarters said, “We heard that 70 elderly people who sustained damage from the tsunami are going to the shelter, and it seems that they are still wet. We order you to take these clothes to the shelter. Anyway, please do what you should do while consulting with the nursing home staff.” After that, I did not receive further instructions from headquarters. Id 02
- The men’s clerks at the local government were continually changing. Until the end of March, we were forced to operate an evacuation center. Id 01

At a large-scale evacuation shelter with approximately 2000 people, daily supplies flowed in a disorderly fashion, and administrative officials, including the PHNs, were overwhelmed by the logistics. The PHNs were unable to assess the individual health problems of the residents in the shelters, although they had some overall idea of the health issues in the shelter.

- The situation [of the shelter] did not to allow us to work only on health issues. I could deal with hearing the story of a person who came [to the place where we were], and I was able to handle it. However, I couldn’t really go around to each room in the shelter and listen to and assess each person’s health problem. Id 03

Many people demanded prescriptions for chronic illnesses but had information only about the color and shape of the drugs, not their names and efficacies.

- When asked, “Did you really take your blood pressure medication?” most of the residents gave vague answers. I think everybody has been given their own prescription note, but they never read the contents exactly. Id 01

The PHNs of the support team could not leave the evacuation shelter to rest until 20 days after the earthquake, when medical teams from non-disaster areas arrived at the evacuation shelter.

##### Patrol Type

Three administrative support teams were immediately created with two PHNs/nurses. These teams performed safety checks at 10 of the 17 shelters in this district, fearing deaths from the second tsunami. After these checks, all members of these teams returned to the branch office to share relevant information. Because the PHNs in this district offered a population-based practice as an ordinary type of routine healthcare, they could identify nurses who worked in nursing homes or at the municipal hospital among the residents in the shelters. Consequently, self-management teams for medical issues developed voluntarily in their respective shelters. After that, the local government officially requested that they provide medical care management for the shelter.

- [The day after the earthquake] we asked the nurses at the shelter—including midwives, nurses from city hospitals, and teachers at the school for the disabled—to watch the residents at the shelter and contact us if something happened. Id 11

Three administrative support teams had to be able to make rounds at each shelter twice daily, at which time they obtained and addressed status reports from the self-management teams on medical issues. Due to the activities of these self-management teams, approximately one week after the disaster, the PHNs were able to take some time off at their own homes.

#### 3.3.3. Theme 3: The Autonomy of the Shelter Residents: The Shelters as Places for Daily Life

The large-scale evacuation shelter had high turnover during the acute phase of the disaster. Because almost all of the residents in the community fled to the evacuation shelters to survive, after the tsunami, they began to go to other shelters to find their families. The characteristics of the evacuation shelters were also changing according to the disaster phase. The residents’ autonomy in the shelters became an important issue as they gradually became settled and the shelters became daily living spaces. Thus, the residents’ autonomy became a new issue.

##### Station Type

The PHNs allocated by the local government had been enthusiastic about operating the shelters from the initial stages, and the residents thought the PHNs should play core roles in the shelters as the most appropriate method of operating them.

- Since we had to operate the shelter from the beginning, the residents naturally thought that only the administrative officials should operate the evacuation shelter. Id 02

However, the PHNs themselves were also very anxious because the response of the operating shelters had to be decided by one person under changing conditions without instructions from headquarters.

- I am a public health nurse, so I had received various consults from everyone and decided on my own. However, I was very anxious because I wasn’t sure if these decisions were appreciate. I had been alone. Id 06
- When local residents made rice balls for shelters, they were asked if the city could afford to serve vegetable dishes. But my first concern was whether these budgets could be implemented. I can’t argue with anyone, I don’t have the right to allow it. Having to decide alone was a big worry Id 01.

The PHNs were not aware of the situations at evacuation shelters other than their own, and while PHNs received supplies from headquarters, each evacuation shelter was independent in terms of information and organizational management policies.

- I had no idea where a colleague in the same department was assigned to the shelter. I didn’t even know that there were 100 shelters in the city until April. Id 03

When the daily supplies began to be distributed regularly within the shelters, the PHNs had discussed the manner of operating the shelters with the residents. As a result, some tasks such as drinking-water management and the cleaning of toilets were left to the residents; however, a residents’ management system had not been organized. The PHNs inferred two reasons for this omission. One was the lack of regional connections, as the residents in the large-scale shelters comprised citizens from different areas. Another reason was that the constant presence of administrative officials increased the residents’ dependence on them.

- I now think that what we had to do was more than manage the evacuation shelters. Certainly, we should have been able to leave the shelter a bit when the medical team arrived. Id 01

Under such severe circumstances, strong human relationships had been established between the shelter residents and the PHNs in a short time. Approximately 10 days after the earthquake, a community medical research team was formed at Kesennuma City Hospital to conduct a health survey of the community, and the PHNs at the evacuation shelters were asked to participate. However, this plan failed. A doctor on this team explained why the PHNs’ participation was unsuccessful: “An unexpected event happened. When we tried to get the PHNs out of the shelter, the shelter residents cried, ‘Don’t take the PHNs away from us.’ The PHNs emotionally and tearfully insisted, ‘We cannot leave these people behind.” The following data illustrated the PHNs’ explanation for their own activities several years later.

- I now think that we should have not felt that a PHN was a member of the shelter as well. The more I stayed in the shelter, the more work I had to do. But we should have acted as members of the local government. Id 01

##### Patrol Type

Because the ordinary human relationships in the community were imported into the evacuation shelters, the shelters were managed by the residents at an early stage of the disaster response. Because the PHNs were not involved in the logistics, they conducted their work as medical professionals with population-based skills. In addition, the chief PHN in the public health department of the branch office combined information from the various shelters in this district. For example, when the self-management team for medical issues identified a resident in need of emergency transport, this team immediately contacted the chief PHN in the branch office and supervised the assessment.

- The teams did not act without notifying the chief clerk of the PHNs in the branch office. This did not mean that the branch office approved these teams; they provided systems for sharing information and behaved in a cohesive manner. Id 08

#### 3.3.4. Theme 4: The Relationship with the External PHN Support Teams: The Disaster Recovery Phase

Approximately 12 days after the disaster, the external PHN support teams began to work continuously within the evacuation shelters. The arrival of these teams was a major turning point for the PHNs in both districts, who were dedicated to the operation of the shelters. The sharing of information with these teams became a new issue because the external PHN support teams essentially replaced all of the team members within one week.

##### Station Type

The external PHN support teams contributed somewhat to bearing the burden of the stationed PHNs’ tasks. First, the external PHN support teams conducted individual medical interviews of the residents, which had been impossible before then. Second, the stationed PHNs were able to return to their homes for the first time and rest. The PHNs’ tasks then changed to sharing the health information of the residents in the shelters with the current external PHN support team and passing it on to the next incoming team.

- From the day when the teams came, the evacuation shelter changed completely. First, I was not able to talk to each resident individually, so I asked them to conduct health interviews for each resident. Id 07
- Since the supporters were replaced in a week, I had to hear the information from the previous teams and convey it to the new team each time. I had to endlessly repeat this information sharing. Id 14
- With the arrival of support public health nurses from non-disaster areas, we were finally able to return home. Id 02

Three weeks after the earthquake, which was the beginning of a new fiscal year, the administrative director of the Kesennuma government commanded the PHNs to leave the shelters and engage in community health activities.

- Until then, my heart was full dealing with the problems at the shelter. When I returned to being an administrative official temporarily, I became aware that what I had to do was turn from the management of the evacuation shelter to solving the community’s health problems. Id 07

However, the Kesennuma government did not have a department in charge of the shelters, so the PHNs spent several hours in the evacuation shelters each business day for management and information sharing with the external PHN support teams.

##### Patrol Type

Two weeks after the earthquake, the medical self-management teams returned to their daily work, and the external PHN support teams arrived at approximately the same time. In this district, the chief PHN in the branch office served as a counterpart to the external PHN support teams. The shared communication between the chief PHN and these teams was conducted at the branch office away from the shelters, and administrative officials other than the PHNs sometimes helped to assign health issues to the external PHN support teams. The evacuation shelter management was transferred smoothly from the medical self-management team to the external PHN support teams while maintaining the patrol approach of the PHNs. The information on individual health issues collected by these teams was useful for considering the next healthcare plan in the region.

- Once I explained to the residents about the beginning of the continuous activities of the external support team, I left the shelter and entrusted the management to them. I was able to integrate the information that the branch government had with the new information captured by the support teams; therefore, together, we could discuss what to do next. Id 08

Furthermore, the withdrawal of the PHNs from the evacuation shelters did not become a major problem, and it was possible for them to shift naturally to engaging in community medical activities.

## 4. Discussion

This study compared two forms of PHN performance observed in two types of shelter based on four themes for the one-month period following a severe disaster. The PHNs of each district supported the residents, who were suffering from both physical and mental damage and who were struggling to develop autonomy concerning the management of the shelters. However, we interpreted stationed PHNs to have had regrets about some of their activities at in the evacuation shelters. Moreover, the need to make decisions alone in operating shelters imposed a psychological burden on PHNs in the Old City.

What we can learn from their experiences is that the concept of operational periods in an ICS should be adapted not only to points of central command but also to small organizations on the frontlines [39]. In the operational period, the incident commander performs its own tasks and thus needs to take turns. This period typically begins 24 h after the incident occurs, and the length of the subsequent periods should be adjusted as incident response activities progress. In adapting this concept, a crisis response should function based on standard organizational operations rather than based on personal competencies. Our data indicated that PHNs in the Old City stayed in evacuation shelters for more than 10 days without a change of clothes, even though other administrative officials worked shifts. According to our data, on the day after the disaster, some PHNs in the Old City had been recalled to the headquarters and reallocated to another evacuation shelter where there were numerous elderly people who had been affected by the tsunami. While not adopted in the operational period, the new target setting was applied to respond to changing conditions. The length of subsequent periods depended on the progress of disaster response; however, we believe that adequate opportunities would have been available when a shelter was changed into a residence or when external PHNs supporters was supplied the shelters.

Human resource allocation, as a key source of support for residents in the community, should be independent of personal factors. In the Old City, territorial connections such as the PHNs’ places of residence were used to allocate PHNs. Well-known PHNs were able to rapidly form strong relationships with residents in the shelters; however, these relationships entailed both strengths and weaknesses. The former was that PHNs’ were able to quickly locate human resources with useful skills among the residents as on medical issues in patrol-type shelters. The latter was that strong relationships made the organization inflexible and led to the refusal to participate in the community’s health survey team. PHNs required a resident management system one week after the disaster; however, it failed to develop. Residents took it for granted that the manager would perform all the work, so it may have been too late to propose self-management by the residents.

We should not fail to take note of the PHNs’ negative interpretations of their actions in our study. The data of Id 01 showed the following self-assessment: “What we had to do was more than manage the evacuation shelters” and “I now think that we should have not felt that PHNs were members of the shelters as well.” PHNs in the Old City had been compelled to decide by themselves under the isolated circumstances with little information or instructions from the headquarters, and they had frequently talked about their anxieties over this decision making. On the other hand, although Motoyoshi district had been isolated from the Old City as well, the PHNs had been able to grasp the full information on the shelters in the district and shared the decision-making process with members of the self-management team on medical issues. In October 2020, nine years after the disaster, a co-author said that the PHNs in Motoyoshi seem to be doing a great job and have a sense of accomplishment. On the other hand, the state of the PHNs in the Old City seems to be different. But there seems to be a tacit understanding that people should not dare to talk. For PHNs, the psychological burden of making independent decisions about small daily events, such as vegetable purchase budgets, can be considerable. This issue should be discussed in future studies. Our study could not clarify the factors associated with negative meaning-making on their own.

Kelin et al. previously indicated that mass evacuations and people with chronic diseases were new challenges in the operation of evacuation shelters due to increasing population densities and aging societies worldwide [3]. Our study showed that these challenges occur not only in highly urbanized large cities but also in relatively small cities. A major anxiety of the evacuees was the lack of medications for chronic diseases. A position paper by the Association of Public Health Nurses [40] suggested that PHNs should not only provide relief activities but also assume leadership in command centers during emergencies, and participate in policy planning. However, importantly, the local PHNs were also disaster victims, even as they enthusiastically fulfilled their roles. Local PHNs may be too dedicated to their professional responsibility to other disaster victims. Under the station-type policy, the PHNs lived continuously in the evacuation shelters and had little rest for nearly 20 days, whereas under the patrol-type policy, they could take a day off at their own homes once per week at a relatively early stage. To operate shelters, an awareness of residents’ behaviors is also crucial. Phillips et al. [29] identified two types of relationship between residents and administrators regarding decision-making management styles after Hurricane Katrina. In the first, a representative of the residents and administrators shared their decisions, while in the second, bureaucratic decisions were made by an executive for the residents. This finding might be similar to the theme of “the autonomy of the shelter residents” in our study. Shared decision-making between residents and administrators is important for identifying residents’ need to inform shelter operations. The station-type policy for operating the shelters did not resemble either of these two relationship types. The stationed PHNs dealt with the shelter residents very enthusiastically; however, their performance was neither organized nor planned. Even if PHNs are stationed at shelters, representatives of the residents should be involved in shelter operations from an early stage. Ironically, due to the shortage of PHN teams, self-management teams for medical issues were created in the patrol-type shelters in our study.

The methodology of the grounded theory approach requires several analytical processes. Such processes include continuous comparison, where initial analyses and data collection are performed in parallel, and theoretical sampling, where the sample subjects are objectively selected to produce better results during the analysis. A continuous comparison based on the objective grounded theory approach by Glaser and Strauss [36] requires that codes should be assigned to all segments of the transcriptions immediately after each interview. Although the 15 datasets used in our study were rigorously coded and categorized, the initial analysis of 79 interviews was coded to generate summaries of the interviews. However, Charmaz, K, who promotes the constructivist grounded theory approach, has noted that overly strict methods may hinder open analysis [41].

We present three findings from this study. First, it is worth considering the adoption of an operational period for small organizations at the frontline of disasters such as shelters. The allocation of the PHNs in the large-scale shelters would have been adequate to grasp the current situation at the onset of this earthquake. However, the information should be collected and evaluated by the headquarters, and then the next mission should be planned. The shelters’ characteristics changed twice during the emergency response phase when they were transformed into a daily living place, and when external PHN support teams began to provide continuous activities. The policymakers of risk governance should not have missed these opportunities. Second, PHNs should locate residents with relevant competencies in shelters from the early stage. Once a situation develops in the shelters where all tasks are handled by administrative officials, the shelter residents might be able to acquire a little autonomy, and PHNs’ awareness needs to be changed not only to operate the shelter on their own, but to encourage residents to manage the shelter. Moreover, educational programs to establish basic civic behavior should be developed as an investment in disaster risk mitigation. Japan has no citizen educational program on DRR planned at the national level; however, other programs for citizens such as the Dementia Intensive Support Program [42] and Self Fire Defense Groups [43] have long histories in training citizen volunteers, who then spread throughout Japanese communities. We think that an educational program for citizens experiencing disasters can be developed using Japanese culture and existing program properties. In terms of personal preparation for DRR, we propose that patients should take pictures of their medications on their mobile phones and email them to their families during the course of their ordinary lives. This will allow patients to obtain accurate prescription data at the shelter relatively quickly. Third, personal factors should be independent of human resource allocation to support residents in the community. When the relationships between administrative officials and shelter residents becomes too strong, it can be an obstacle to reallocating administrative officials and ensuring the appropriate operation of shelters. Human resource allocation is one of the most important and limited resources during a public health emergency

## 5. Limitations

This study has some limitations. First, the sampling strategy using a gatekeeper with personal networks in this region might have caused a bias. However, in an irregular situation under the conditions of a severe disaster, feasibility was the priority. Second, we obtained only limited data from 10 large-scale shelters out of the 65 shelters in the Old City. We assume the other shelters were of either the patrol or station type; however, their details are unknown. Third, damages after disasters vary widely according to the disaster type, so the present findings should be applied to other situations carefully. Water damage from hurricanes is predictable, whereas earthquakes and tsunamis are sudden and unpredictable. Even earthquakes vary in Japan, for example, a building collapse caused massive casualties in the earthquake disaster in Kobe in 1995. Meanwhile, the damage from the present earthquake disaster was mainly tsunami-related, and survivors were not physically injured. Therefore, the major anxiety of the evacuees was the lack of medication for chronic diseases. Expected roles for PHNs should be considered according to the situation.

## 6. Conclusions

We identified two operating policies by PHNs in the evacuation shelters—the station and patrol types—and compared them using four themes. Human resource allocation constitutes a key source of support for residents in the community after a disaster. In emergency situations, operational periods would be meaningful for the relatively small organizations on the frontline. Even if emergency situations need some PHNs assigned to shelters continuously, the response headquarters will need to collect information on a short-term basis and update the plan according to the response progress, while maintaining the standard mechanism of the evacuation shelter organization.

## Figures and Tables

**Table 1:** Demographic data from before the Great East Japan Earthquake and the numbers of damaged houses and evacuation shelters in the three districts.

District	Old City *	Karakuwa	Motoyoshi
Area (km^2^)	130.03	42.31	106.7
Population **	52,089	7420	10,855
Percentage of population over 65 years old **	29.7%	34.2%	30.8%
Population density (person/km^2^) **	145.3	175.4	101.7
Damage Status			
Houses			
Fully destroyed	11,391	1867	2417
Destroyed, difficult to live in	1689	80	289
Partly destroyed	2666	186	558
Percentage of all houses	38.40%	28.20%	28.50%
Evacuation shelters			
Maximum number of shelters	65	16	17
Maximum number of residents in the shelters	2000	230	530
Total number of residents in the shelters	14,491	1250	3005

* Excluding the island of Oshima. ** Current as of December 1, 2010. [33,34].

**Table 2:** Narrative data from the four themes and the two operational policies.

Operational Policy of the Evacuation Shelter	
Station Type (in the Old City)	Patrol Type (in Motoyoshi District)
Theme 1: The lack of an operational preparedness policy for the shelters	
Everyone gathered in the city office’s large hall, and, while still not knowing anything at all about the disaster conditions, we received a command from Headquarters to move to an evacuation shelter. We were told to scatter to each of the evacuation shelters. Id 02	For my title, I was a leader. Since the section manager happened to be there, I said, “Section manager, no one can stay in the evacuation shelters. We must move between the shelters in two-person teams,” and with that, a patrol-focused evacuation shelter management style was established. Id 11
So then, we wondered, “When we do go out there, what should we do?” Id 01	With administrative nurses, there are two persons, and with public health nurses, there are four persons. I thought this could not cover all of the evacuation shelters. I recommended the following to the community health policy leader: “Divide into groups of two nurses and rotate around the evacuation shelters to check the situation.” Id 09
Since we did not know who would be coming to the evacuation shelter, the management varied completely, depending on the evacuation shelter. Id 01	I thought that fixed placement was impossible because there were few public health nurses. It’s been like that from the first day of the disaster. Id 09
Theme 2: The provision of support to the shelter residents	
At the large evacuation shelters, with everyone coming together without territorial connections or links, since the shelters had the presence of an administrative staff, everything that has to do with sheltering is done by the administrative staff. Id 13	There were the municipal hospital nurses and nurses who were working independently, among whom leadership was just naturally generated. Id 04
The evacuation shelter administrative operations involved the delivery of meals, the creation of name lists, and the carry-in of resources, and it ended with that. Since I was a medical technician, I could not, of course, say that I couldn’t handle it. Id 02	The day after the earthquake, we asked the nurses at the shelter—including midwives, nurses from city hospitals, and teachers at the school for the disabled—to watch the residents at the shelter and contact us if anything happened. Id 11
I couldn’t do it (nurse work), because there was no other person. The situation did not allow us to work only on health issues. Id 03	Of course, it was not possible for health professionals to be stationed at all of the places where the shelters were established, so I checked the situation by walking around the places that became shelters in order, with one pair of two health professionals. Id 09
I remained at that evacuation shelter all the way until around April. With the arrival of the support public health nurses from non-disaster areas, we staff were finally able to return home. Id 02	
Theme 3: The autonomy of the shelter residents	
There were some small evacuation shelters where such residents’ associations moved. A large evacuation shelter where everyone gathered from various places was managed by the administration. There are administrative staff here as well, so the administrative staff must act in the core role of evacuation shelter management. Id 10	Self-nursing teams had been created. These team members were nurses who couldn’t go to work, and they checked on the residents’ health in the shelters while we were patrolling. Id 04
We talked about our shelter several times (about self-government management). A problem has arisen among us that said, “If possible, people who are evacuating should be able to operate independently.” Was it about a week? Id 01	From the next day, an independent nursing team consisting of midwives, municipal hospital nurses, and elderly home nurses cared for and evaluated the health problems [of the residents] at the evacuation shelters. Therefore, I requested health management at the shelter. I asked them. “Please contact me immediately when any accidents occur.” Id 11
Every morning a staff member holds a meeting and says, “Let’s do this today.” Since we had to operate the shelter from the start, the residents naturally though that only administrative officials should operate the evacuation shelter. Id 02	The teams did not act without notifying the chief clerk of public health. This did not mean that the branch office approved these teams; they provided systems for sharing information and behaved in a cohesive manner. Id 08
I now think that what we had to do was more than managing the evacuation shelters. Certainly, we should have been able to leave the shelter for a bit when the medical team arrived. Id 01	
Theme 4: The relationship with the external PHN support teams	
Since the supporters will be replaced in a week, I have to hear the information from the previous teams and convey it to the new team each time. I have to endlessly repeat this information sharing. It’s a new support form for the shelter. I stayed in the shelter for half a day (even in April). I only handed over the information and then worked in the area as a public health nurse. Id 14	Once I explained to the residents about the beginning of the continuous activities of the external support team, I left the shelter and entrusted the management to them. I was able to integrate the information that the branch government had and the new information captured by the support teams; therefore, together, we could discuss what to do next. Id 08
If I knew that support was coming at that time, I might have left the shelter early. But by staying (we are in the shelter) for a long time, it was rather hard to leave the shelter. Id 01	Since information was collected in only one building (the branch office), it was easy for the public health nurses to have external support. After that, we were able to make allocation plans for support with other members. Id 11
At that time, I first noticed that my original work had stopped. I got back to my heart. It’s April, and I have to do a lot of things, but it’s all stopped. Id 02	We were able to integrate the information held in the city office with the new situation captured by the external supporters. So I was able to think together with the support team about what to do next. Id 04

## Appendix A

**Table A1:** Interview guides of the first phase for professionals in the medical and welfare fields and administrative officials.

Please Share in Detail What Happened Immediately after the Disaster.
What did you do for the first 72 h after the disaster?
What changes occurred after 72 h?
Please tell us about your dilemma as a victim and your professional responsibilities.
Please share any lessons concerning disaster recovery.
